# Plan for the Better Handling of Venereal Diseases in the Army

**Published:** 1919-01

**Authors:** 


**Plan for the Better Handling of Venereal Diseases in the Army.** By Major Loyd THOMPSON, M. R. C. *The Military Surgeon*, August, 1918.

The author insists upon the necessity of treating venereal disease at the earliest possible moment after infection. He proposes that a " venereal dispensary " be attached to every division and that each regimental infirmary be equipped for treatment of acute and chronic gonorrheal urethritis and chancroid. This dispensary should consist of the following :

(1) A laboratory, completely equipped for the diagnosis of all venereal diseases, including apparatus for dark field examination, and Wassermann test.

(2) Treatment room for syphilis, fully equipped for the administration of many doses of salvarsan daily.

(3) Treatment room for gonorrhea, including apparatus for treating gonorrheal arthritis.

(4) Record room.

(5) Store room, containing a supply of drugs and chemicals for 6 months. (The salvarsan to be supplied monthly).

Personnel of the dispensary. (1) Director of venereal diseases; (2) 4 captains or lieutenants; (3) 9 enlisted men as laboratory assistants, clerks, etc.

The director and his staff should make lectures and demonstrations on the methods of handling venereal diseases; venereal inspection of men should be held daily in each company; all cases of genital sores should be reported at once to the dispensary for diagnosis. Cases of acute gonorrheal urethritis should report to the dispensary twice daily for 3 days, after which they should be sent to the regimental infirmary for further treatment, and referred again to the dispensary for examination when the regimental surgeon considers them cured.

The same applies to cases of uncomplicated chancroid.

Cases of syphilis should be required to report to the venereal dispensary for salvarsan treatment on the day following diagnosis, and at specified periods for Wassermann tests, as ordered by the syphilologist.

All cases of venereal disease with severe complications, as well as cases of disease of the genito-urinary organs, other than the venereal, and cases of diseases of the skin other than syphilis, should be sent to the base hospital for treatment.

By this plan all cases of venereal diseases should be detected and treated in very early stages, which would result in a large percentage of cures.

